# Experimental Study on Spread and Burning Characteristics of Continuous Spill Fire Leaked from a Point Source under Different Slopes

**DOI:** 10.3390/ijerph20054323

**Published:** 2023-02-28

**Authors:** Xiaoxiao Sun, Hong Huang, Jinlong Zhao, Xiang Zhang, Guangheng Song

**Affiliations:** 1 Institute of Public Safety Research, Department of Engineering Physics, Tsinghua University, Beijing 100084, China; 2 School of Emergency Management and Safety Engineering, China University of Mining and Technology, Beijing 100084, China

**Keywords:** spill fires, point source, slope, burning rate, flame height, heat loss

## Abstract

Liquid fuel is widely used in industry and transportation. Liquid fuel leakage usually results in some spill fire accidents. In this paper, the effect of slope on the spread and burning behaviors of continuous spill fire from a point discharge source was studied by experiments. The flame spread rate, burning rate, heat convection at the bottom surface, flame feedback radiation, and flame height were analyzed. The results show that the spread area has an increasing trend with the slope, and the length of the spread area increases obviously, while the width of spread area shows an opposite trend. Moreover, the burning rate and the flame height of the steady stage decreases significantly with the slope increase, which can be attributed to the increase of heat convection between the fuel layer and bottom for the larger slopes. Subsequently, a burning rate model for the steady stage is built considering fuel layer heat loss and validated by the current experimental data. This work can provide guidance for the thermal hazard analysis of liquid fuel spill fires from a point source.

## 1. Introduction

Liquid fuel leakage and spill fire accidents usually occur during liquid fuel storage, transportation, and processing. Compared with pool fires and thin liquid fuel layer burning, spill fire has a larger burning area and is difficult to extinguish due to the spread process of the liquid fuel layer, which poses a great threat to the safety of fuel storage and transportation [1,2]. Moreover, the spread and burning process of spill fires are sensitive to the change of slope. Even a small slope will have a great impact on how the spill fire disaster develops. For example, a serious spill fire accident caused by an oil tank truck rolling over and oil leaking out occurred in 25 July at Chengdu of Sichuan Province, China [3]. It was reported that the leakage of oil from the truck caused a spill fire with huge burning area and an extended traffic block on a sloping road surface. As a result, it is important to study the characteristics of continuous liquid fuel spill fire under different slopes.

Scholars have studied the spread and burning behaviors of spill fires. Mealy, Benfer and Gottuk et al. [4,5] analyzed the effects of spread area, spread bottom surface, spread thickness and other factors on the burning rate of continuous spill fires through experiments about the spread and burning process of gasoline, heptane, kerosene, and ethanol on concrete, steel plate and fire blanket. They put forward the calculation formula of spill fire burning rate through the correction of the pool fire burning rate. Ingason et al. [6] presented a spill fire experiment in a tunnel, which included different leakage rates, leakage types, and heat release rates on sloping surfaces. They found that the heat release rate of spill fires is about 1/3 to 2/5 compared with a pool fire with same size. They also presented models for estimation of leakage rates, spillage sizes, and heat release rates for different scenarios. Li et al. [7,8] conducted some continuous n-heptane spill fire experiments on a water surface in a rectangular trench (L: 12 m; W: 1 m). In their study, a spread model was modified to simulate the spread process based on the balance between viscous forces and gravity. Zhao et al. [9,10] carried out a series of large-scale continuous spill fire experiments on rectangular fireproof glass (L: 6 m; W: 0.8 m). They divided the whole spread process into five phases: spread burning, shrink burning, quasi-steady burning, boiling burning, and extinguished. In addition, they found that the burning rate of the quasi-steady burning phase is lower than that of pool fires under the same burning scale. Li et al. [11,12] studied the spread and burning laws of a continuous spill fire in a tunnel (L: 12 m; W: 1 m; H: 1 m). They found that tunnel environments enhance the burning intensity and radiation heat penetration and built a semi-empirical model to predict the burning area of spill fires in tunnel environments. Pan et al. [13] conducted a systematic experimental analysis of continuous n-butanol spill fires. They found that the sum of sensible heat and convective heat loss accounts for more than 80% of the total heat flux of continuous n-butanol spill fires. These studies focus on two continuous spill fire scenarios: spill fires leaked from a point source, and spill fires leaked from a liner source. However, regarding grounded continuous spill fires, the previous studies only address continuous spill fires that originate from a liner source, and few works address spill fires that originate from a point source. Moreover, the above analyses find that the whole spread process can be divided into multiple stages, and that the burning rate at the quasi-steady burning stage is lower than that of pool fires under the same burning size. However, the slope effect is not considered in these studies, even though this parameter has a great influence on the spread and burning behaviors in real-life accidents.

In recent years, the slope effect on the spill fires has attracted some scholars’ attention. Li et al. [14] studied the spill fire on a sloping stainless-steel trench with slopes of 0~4°. They found that the maximum spread area was sensitive to the slope, while the steady burning area kept constant with the increased slope. Liu et al. [15,16,17] studied the n-heptane spill fire phenomenon under variable slope conditions. They found that the spread rate and burning rate of the spill fire decreased with slope, and provided a simple empirical model of the burning rate. Li et al. [18] studied the unsteady burning behavior of n-butanol steady-flow fire and studied how spread rate and flame height of an n-butanol flow fire behaved for a slope varying from 1~4°. They found that the spread rate of n-butanol increased by 40.8% when the slope increased from 1° to 4°, and the spread front shows a “jump-crawl-retract” phenomenon through the whole steady spread process. Men et al. [19] measured the thickness of the fuel layer at the steady stage of continuous spill fires on a glass surface at 0° and 0.5° using ultrasonic distance meters. They found that the fuel layer thickness of spill fires on an inclined surface is significantly less than that on the flat surface. The fuel layer thickness of spill fires on an inclined surface is about 60% of the thickness of fuel layer on the flat surface. Li et al. [20] studied the spreading and burning process of spill fire under a vertical slope. The results showed that even under a vertical slope, most of the spreading and burning regulations of spill fire can still be applied. A spreading and burning model of spill fire under vertical slope was established. Based on the above analysis, it can be seen that the slope has a great effect on the flame height and burning rate. However, these works mainly focus on the spreading and burning process of continuous spill fire leaked from a liner source. There are few works that have analyzed the slope effect of continuous spill fire leaked from a liner source, and the spread and burning characteristics of continuous leakage spill fires from a point source under different slopes is still not clear. Specifically, heat transfer processes such as convective heat and radiation transmission has not been studied in detail.

This work aimed to analyze the slope effect on the spread and burning characteristics of continuous leakage spill fires from a point source. A series of continuous leakage spill fire experiments from a point source were carried out on a glass platform. The real time burning area, burning rate, and flame height were measured and analyzed. Moreover, the slope effect on the heat transfer process between the fuel layer and the glass platform was discussed. Subsequently, a burning rate model for continuous leakage spill fires from a point source under different slopes was developed.

## 2. Experimental Setup

**Figure 1:**
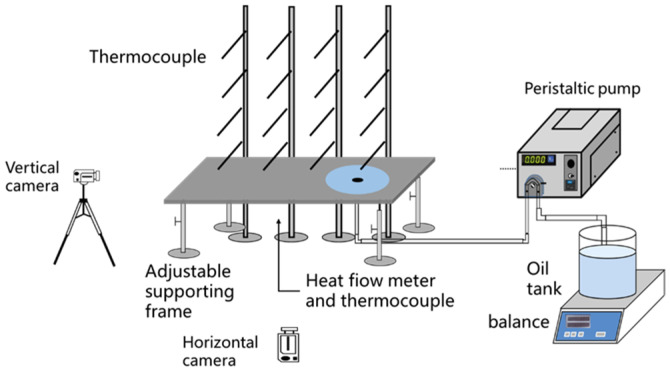
Diagram of experimental device.

As depicted in Figure 1, a variable slope point source continuous leakage spill fire experimental device was used to study the characteristics of spill fire under different slopes. The glass platform is 1.5 m long, 0.6 m wide. A 4 × 4 thermocouple array is placed above the glass platform to measure the flame temperature. The thermocouples type is K, the range is −100 °C~1200 °C, the accuracy is 1 °C, and the sensitivity is less than 1.5 °C. The distance between adjacent thermocouples is 25 cm, the lowest thermocouple is 5 cm away from the glass platform, and the group on the edge is directly above the fuel outlet. 4 thermocouples are placed underneath the glass platform at a distance of 5 cm, 25 cm, 50 cm, and 75 cm from the fuel outlet. These 4 thermocouples are used to measure the temperature of the glass platform bottom. 4 heat flux meters are placed under the glass platform at a distance of 5 cm, 25 cm, and 75 cm away from the fuel outlet. The range of heat flux meters is 0~50 kW/m^2^, the accuracy is 0.1 kW/m^2^, and the margin of error is less than 7%. These heat flux meters are used to measure the flame feedback radiation transmission. All thermocouples and heat flux meters are placed on the central axis of the glass platform. The slope of the glass platform can be adjusted through the adjustable supporting frame, and the glass slope can be measured through the gradiometer with an error of less than 0.05 degrees. The spread process is recorded by the camera, and the variation of the spread distance of spill fire with time is obtained by analyzing the image. The resolution of the camera is 1920 × 1080, and the frame rate is 25 Hz.

In the experiments, n-heptane was used as fuel, and the peristaltic pump was used to deliver fuel at the delivery rates of 30 rpm, 40 rpm, 50 rpm, and 60 rpm. A peristaltic pump can provide a stable flow with a high accuracy. The corresponding relationship between peristaltic pump rotation speed and discharge rate is shown in Table 1. In the experiment, a peristaltic pump was used to deliver n-heptane from the oil tank to the glass platform. A balance is used to measure the weight changes of the oil tank to determine the actual delivery rate. The range of balance is 0~35 kg, the accuracy is 0.01 g, and the sampling frequency is 1 Hz. The slopes of 0°, 0.5°, 1°, 1.5°, and 2° were selected for the experiments.

The overall test conditions are shown in the Table 2.

## 3. Results and Discussion

### 3.1. Analysis of Spill Fires under Non-Ignited Conditions

**Figure 2:**
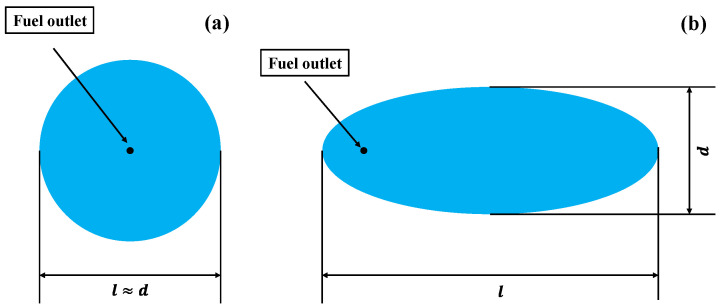
Approximate shape of spread area under non-ignited conditions. (**a**) On the flat surface; (**b**) On the inclined surface.

As it is difficult to directly measure the burning area, two cameras were adopted to record the maximum spread length *l* and maximum spread width *d* of the flame(m). Then, the approximate calculation is determined according to the approximate shape of the spread area in the actual burning process. Figure 2 shows the shape of the spread area under non-ignited conditions. For the flat surface, the burning area in the stability stage can be considered circular (Figure 2a), in which the measured result is *l* ≈ *d*. The burning area can be calculated according to Formula (1):

(1) $$S=\pi {\left(\frac{l+d}{4}\right)}^{2}\;.$$

For the tests with the inclined surface, the shape of the spread area under the non-ignited condition is approximate to an ellipse (Figure 2b). The burning area can be calculated according to Formula (2):

(2) $$S=\frac{\pi ld}{4}\;.$$

**Figure 3:**
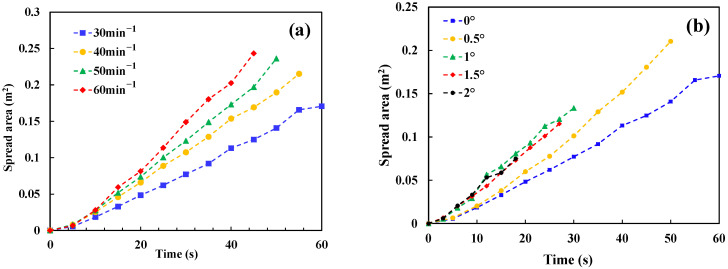
(**a**) Relationship between spread area and flow rate for the flat surface; (**b**) The spread areas as function of the discharge times for the tests with the same discharge rate (30 rad/min).

Under the non-ignited condition, the spread areas of fuel with different discharge rates and slopes are shown in Figure 3.

It can be seen from Figure 3 that in the case of non-ignited conditions, the spread area will not only increase with the increase of discharge rate, but also with the increase of slope. When the slope rises from 0 to 1°, the spread rate increases significantly, but when the slope continues to increase to 2°, the change of spread area is not as obvious.

**Figure 4:**
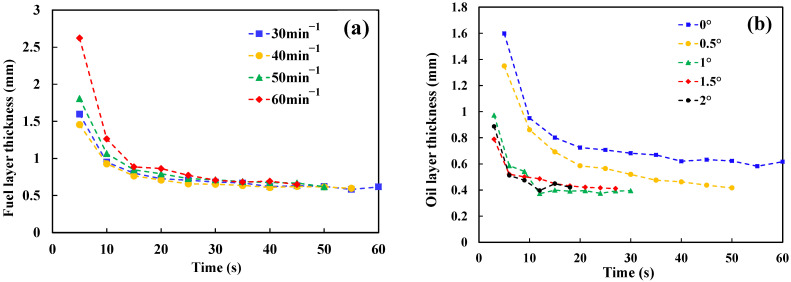
(**a**) Fuel layer thickness as a function of discharge times with a slope of 0°; (**b**) Fuel layer thickness as a function of the discharge times with the same discharge rate (30 rad/min).

Under non-ignited conditions, we assume that the fuel layer thickness during the spread is uniform. The fuel layer thickness during spreading can be calculated through volume conservation, as shown in Figure 4.

It can be seen in Figure 4 that the fuel layer thickness rapidly decreases to a stable value *h_m_* after the beginning of spread, which is independent of the leakage rate, but related to the slope. The *h_m_* under different slopes is shown in Table 3.

**Figure 5:**
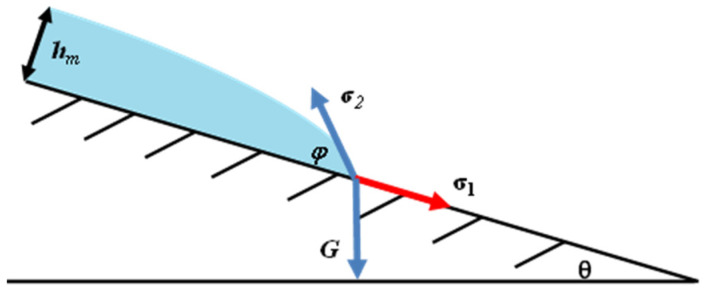
The force analysis when the fuel spreads on an inclined surface.

It can be inferred from Figure 4b and Table 3 that when there is a slope, *h_m_* is significantly less than 0°, but the change of *h_m_* with the slope is not obvious. That means that when there is a slope, the thickness of the fuel layer becomes thinner in the spread process, and the greater the slope, the less time it takes for the fuel layer to be reduced to *h_m_*. This phenomenon can be attributed to the effect of gravity. Hussein et al. [21] gave a formula to calculate the minimum fuel layer thickness *h_m_* using the balance between gravity and the surface tension of the fuel layer:

(3) $$h_{m}=\sqrt{\frac{2\sigma \left(1-\cos\varphi \right)}{\rho g}},$$

where *σ* is the surface tension (N/m), *φ* is contact angle, *ρ* is the density of the fuel layer (kg/m^3^), and g is the gravity constant (9.8 N/kg). For a flat surface, Formula (3) fitted the experimental results well. However, this formula cannot calculate the fuel layer thickness on an inclined surface. Figure 5 shows the balance of gravity and surface tension at the front of the fuel spread. σ_1_ is the surface tension towards the surface, σ_2_ is the surface tension between fuel and air, and G is gravity. With the increase of slope, the vertical component of gravity decreases, and the forward component of gravity increases. To balance the gravity, the vertical component of σ_2_ decreases and the forward component of σ_2_ increases. These changes cause the smaller contact angle *φ*. According to Formula (3), the minimum fuel layer thickness *h_m_* becomes smaller when the slope increases.

### 3.2. Phenomenon of Spill Fires under Ignited Condition

**Figure 6:**
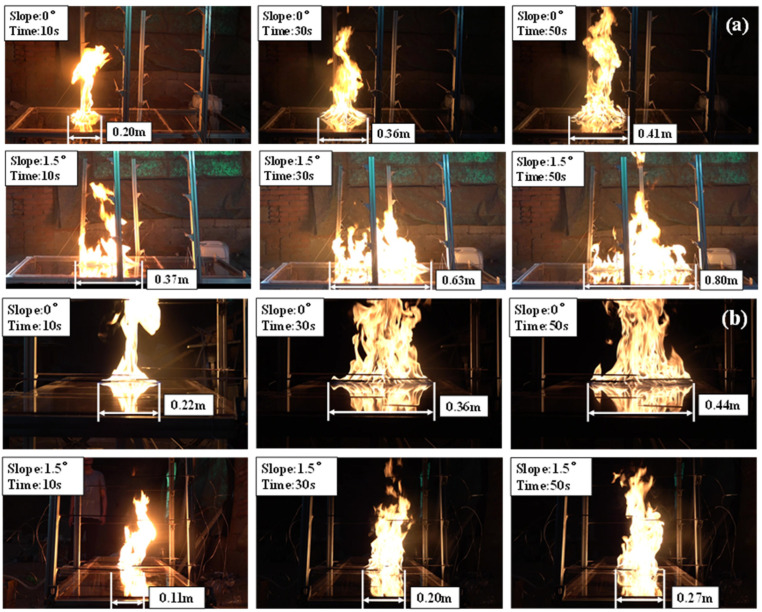
Experimental phenomenon of spill fire under different slopes. (**a**) Record from side direction. (**b**) Record from front direction.

During the experiment, the fuel is ignited immediately when n-heptane flows out of the fuel spill outlet. Then, the spill fire will begin to flow in a downhill direction. The experimental phenomena observed from the side and front are shown in Figure 6.

**Figure 7:**
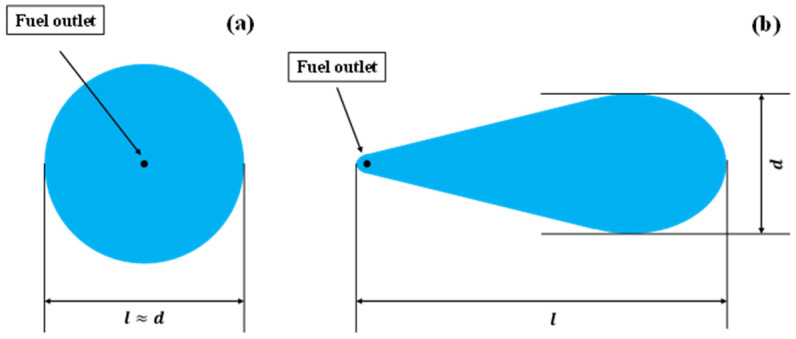
Approximate shape of spread area under ignited conditions. (**a**) On the flat surface; (**b**) On the inclined surface.

On the flat surface, the shape of the spread area is similar to a circle as shown in Figure 7a. The spreading length *l* is approximate to the spreading width *d*. At the beginning of the burning process, the spreading length *l* increases rapidly. However, as the spreading continues, the spreading length reaches the maximum. Considering that the burning rate and fuel consumption increases as the burning area increases, we can conclude that the discharge rate equals to the fuel consumption during this period. At this steady stage, a dynamic balance is established, and the burning area keeps a distance. This phenomenon is similar to those in previous studies [7,9]. On the inclined surface, the shape of the spread area is similar to a water droplet, as shown in Figure 7b. Similar to the spreading process on the flat surface, the spreading length *l* and spreading width *d* increases rapidly at the beginning of burning process. Then, the trend of spreading slows down and reaches a steady stage. Compared to the flat surface, the spreading length *l* is significantly longer and spreading width *d* is shorter on the steady stage.

**Figure 8:**
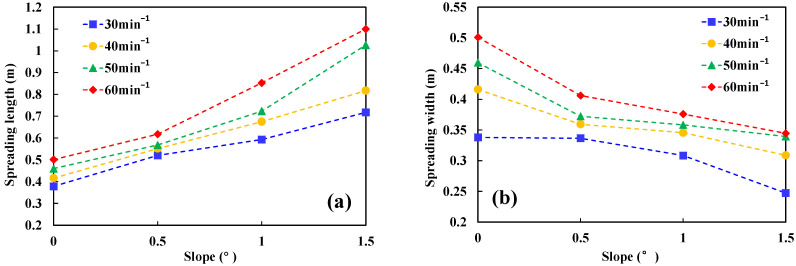
(**a**) The spreading length *l* on steady stage with different slopes. (**b**) The spreading width *d* on steady stage with different slopes.

Figure 8 shows the spreading length *l* and the spreading width *d* on a steady stage with different slopes. When the slope increases, the spreading length *l* becomes longer, but the spreading width *d* shows an opposite trend. This trend is consistent to the non-ignited condition. Because the fuel layer becomes thinner with slopes, the effect of surface tension results in the shrink of spreading width. Based on the approximate shape of the spread area in Figure 7, the spread area can be calculated by Formula (4).

(4) $$S=\frac{1}{2}ld-\frac{1}{4}d^{2}+\frac{1}{8}\pi d^{2},$$

Table 4 shows the spread area with a different slope and discharge rate. For the convenience of calculation, the area at the end of the shrink stage is selected as the burning area in the stable stage. Obviously, with the same discharge rate, the spread area becomes larger with slopes. Considering the dynamic balance between the discharge rate and fuel consumption, it can be concluded that the burning rate of spill fires decreases as slope increases. More detailed discussions about this phenomenon will be in the following sections.

### 3.3. Flame Height under Ignited Conditions

The flame height directly impacts the feedback radiation in the burning process, and is an important part to analyzing the burning process. During the burning process, the flame height fluctuates violently. Zukosiki [22] defined the flame height by introducing the flame intermittent rate. *I*(*H*) is defined as the proportion of time when the flame height is higher than h in the total time among all flame heights. In statistics, then, the average flame height *H* meets the requirements of *I*(*H*) = 0.5. Taking the average flame height as the measurement method of flame height, McCaffrey [23] gives the calculation method of flame height:

(5) $$\frac{H}{D}=3.7{\dot{Q}}^{*}{}^{\frac{2}{5}}-1.02,$$

where *D* is the equivalent burning diameter(m), ${\dot{Q}}^{*}$ is the dimensionless heat release rate, and is defined as

(6) $${\dot{Q}}^{*}=\frac{\dot{Q}}{\rho_{\infty }c_{p}T_{\infty }\sqrt{gD}D^{2}},$$

where $\dot{Q}$ is the total heat release rate (kW/m^2^), which is directly proportional to the burning rate. It can be calculated by Formula (7).

(7) $$\dot{Q}=m^{\prime }H_{c},$$

**Figure 9:**
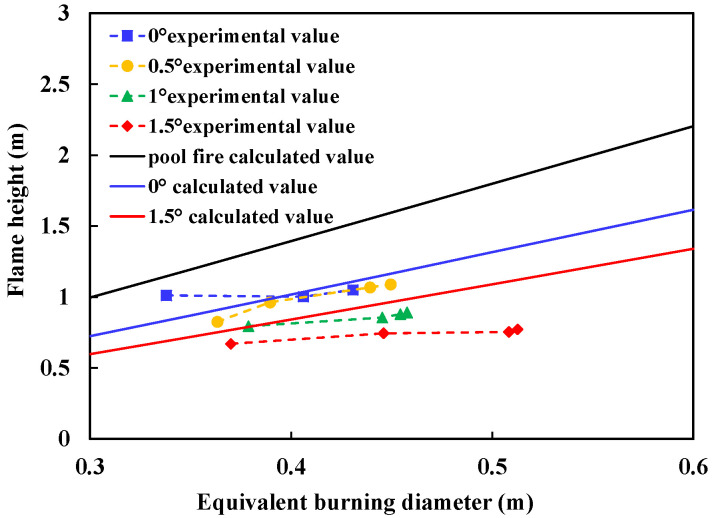
Comparison of Flame Height and Calculated Value.

In the case of slope, the change of average flame height with slope is shown in Figure 9. It can be seen from Figure 6 that if only the change of burning rate with slope is considered, the theoretical value of flame height is noticeably higher than the experimental results. That is, in addition to the change of burning rate with slope, there are other influencing factors affecting the flame height. One obvious factor is the spread pattern of the spill fire. In the case of no slope, the spread surface is circular, and the flame shape is close to conical. In the case of slope, the spread area gradually becomes slender with the increase of slope, and the change of burning area will lead to flame bifurcation, resulting in the decrease of flame height.

### 3.4. Burning Rate under Ignited Conditions

Under the ignited condition, during the burning process in the steady stage, the fuel consumption and discharge are in dynamic balance. At this time, the burning rate can be calculated through the burning area and discharge rate in Formula (8).

(8) $$w=\frac{Q}{A},$$

where *w* is the burning rate(mm/s), *Q* is the discharge rate (mL/s), and *A* is the burning area(m^2^). Thus, burning rate in the steady stage can be calculated as shown in the Table 5.

As seen from Table 5, the burning area gradually increases and the burning rate shows an opposite trend with the slope. The burning rate at 1.5° is about 20% lower than that at 0°, which is in accordance with the phenomenon observed in the experiment of Li et al. [7].

**Figure 10:**
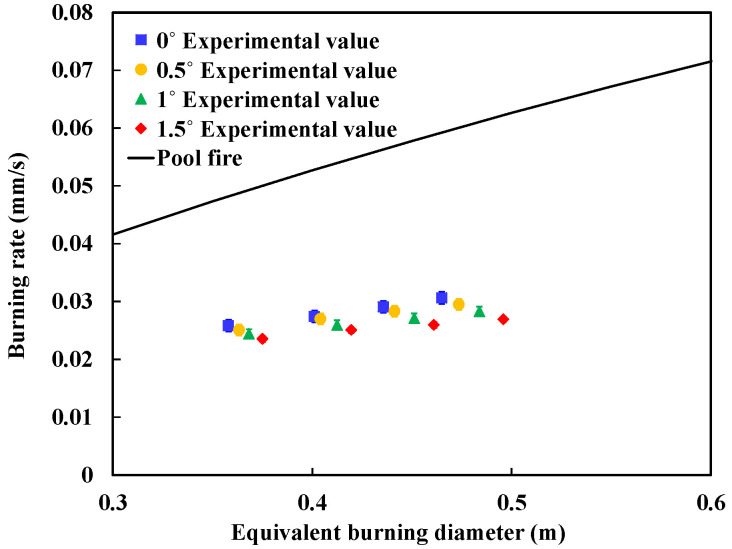
Comparison of burning rate between spill fire and pool fire.

For the burning rate of pool fire, Burgess et al. [24,25] proposed an empirical formula based on experiments:

(9) $$m^{\prime }={m^{\prime \prime }}_{\infty }(1-e^{-k\beta D}),$$

where ${m^{\prime \prime }}_{\infty }$ is the burning rate under ideal conditions (mm/s), *kβ* is the extinction coefficient (m^−1^), and *D* is the equivalent burning diameter (m). For the burning process of n-heptane, the changes of burning rate under different slopes in the experiment are shown in Figure 10.

As shown in Figure 10, we can find that the burning rate of spill fire is significantly smaller than the pool fire with same burning area. This result is in accordance with findings from previous studies [7,10]. Moreover, when the slope increases, the burning area becomes larger and burning rate becomes smaller.

### 3.5. Heat Transfer Analysis under Ignited Conditions

**Figure 11:**
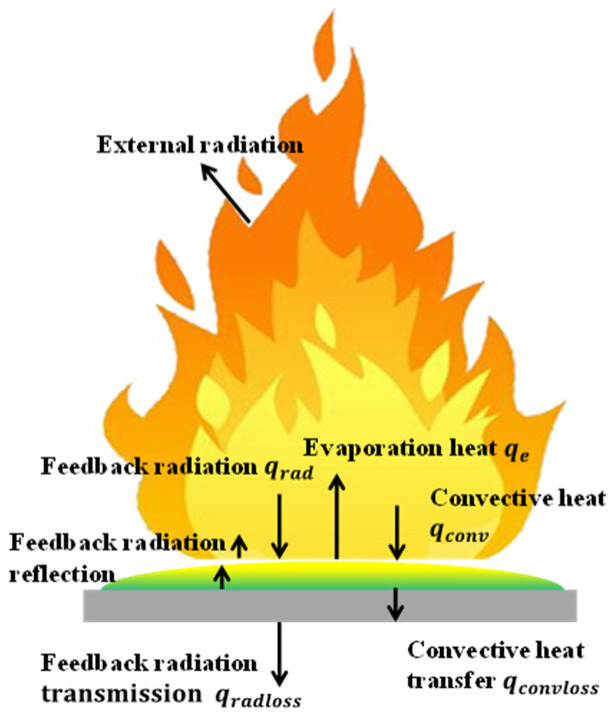
Heat transfer process of spill fire.

It is generally believed that the burning rate depends on the flame heat feedback to the fuel surface for pool fires. The heat loss of the liquid fuel layer is ignored in pool fires. However, this part cannot be ignored in the spill fires because of the thin layer [10]. Figure 11 shows the heat transfer process of spill fires.

In the steady spread stage, based on the heat transfer balance, the heat transfer can be expressed as:

(10) $$q_{e}=q_{rad}-q_{covloss}-q_{radloss}$$

where $q_{e}$ is the evaporation heat of fuel (kW/m^2^), $\;q_{rad}$ is the feedback radiation (kW/m^2^), $q_{covloss}$ is the convective heat transfer loss (kW/m^2^) and $q_{radloss}$ is the feedback radiation transmission loss (kW/m^2^). Evaporation heat of fuel $\;q_{e},$ can be expressed as:

(11) $$q_{e}=m^{\prime }H_{V},$$

where $m^{\prime }$ is the burning rate of the fuel (g/s), and $H_{V}$ is evaporation heat of fuel (kW/(m^2^·kg)). According to the model of Zhao et al. [7], feedback radiation can be expressed as:

(12) $$q_{rad}=\sigma F\varepsilon \left(T_{f}^{4}-T_{a}^{4}\right),$$

where σ is the Stefan–Boltzmann constant (5.67 × 10^−8^ W/(m^2^K^4^)), *F* is the angle parameter, ε is the radiant emissivity of the flame, and $T_{f}^{\;},\;\;T_{a}^{\;}$ are the temperature of the flame and environment (K). Radiant emissivity of the flame ε can be expressed as:

(13) $$\varepsilon =1-e^{-kL},$$

where *k* is the extinction coefficient, and *L* is the effective thickness of gas to fuel layer surface. *L* is proportional to the combustion diameter of the pool fire and can be expressed as:

(14) L=βD,

In pool fires with a radius greater than 0.3 m, the feedback radiation mainly controls the burning rate of the fuel. The burning rate of the fuel can be calculated as:

(15) $$m^{\prime }=\frac{q_{rad}}{H_{V}}=\frac{\sigma F\left(T_{f}^{4}-T_{a}^{4}\right)}{H_{V}}\left(1-e^{-k\beta D}\right)={m^{\prime \prime }}_{\infty }\left(1-e^{-k\beta D}\right),$$

This result is Formula (8). In the spill fire, the burning rate of spill fire can be expressed as

(16) $$m^{\prime }=C_{\delta }{m^{\prime \prime }}_{\infty }(1-e^{-k\beta D}),$$

where $C_{\delta }$ is the burning rate ratio. Combining Formulas (10), (11) and (15), $C_{\delta }$ can be expressed by the following formula:

(17) $$C_{\delta }=1-\frac{q_{covloss}+q_{radloss}}{q_{rad}},$$

where $q_{rad}$ is the total feedback radiation (kW/m^2^). Zhao et al. [10] proposed that the feedback radiation transmission of the n-heptane thin fuel layer pool fire can be expressed by the following formula through their experimental study on the burning process of thin fuel layer pool fires:

(18) $$q_{radloss}=0.8q_{rad}e^{-ah}+0.2q_{rad},$$

where *h* is the fuel layer thickness (mm), and *a* is the absorption coefficient (mm^−1^), which is $a=0.55{\;\mathrm{mm}}^{-1}$ in the process of n-heptane pool fire burning. When the $q_{covloss}$ and $q_{radloss}$ is measured, the burning rate can be calculated by Formulas (16) and (17).

#### 3.5.1. Heat Convection between Fuel Layer and Bottom Surface

**Figure 12:**
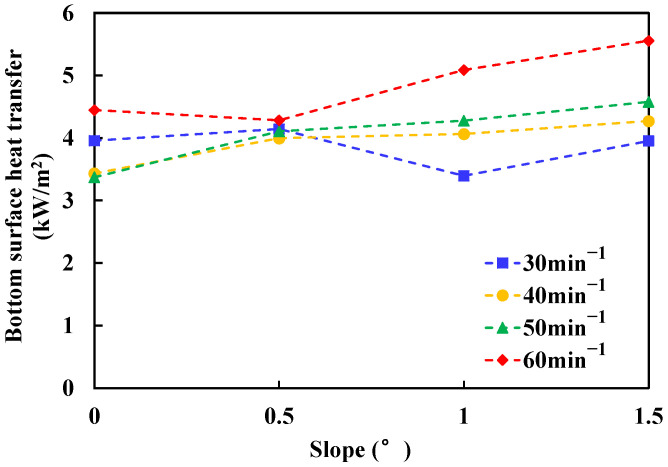
Variation of flow heat transfer of n-heptane spill fire at bottom surface with slope.

In this experiment, without considering the radiation absorption of the glass bottom, the convective heat transfer of the fuel layer to the glass can be calculated by the following formula [10]:

(19) $$q=Ac\rho \overset{h_{g}}{\underset{0}{\int }}\frac{dT}{dt}dh,$$

where *A* is the burning area (m^3^), *c* is the specific heat capacity of the glass (kJ/(kg·K)), *ρ* is the glass density (kg/m^3^) and $h_{g}$ is the glass thickness (mm). *T* is the temperature at the position in depth *h*(K). For the convenience of calculation, it is assumed that the upper surface temperature of the glass is the boiling point temperature of the fuel layer $\;T_{m}$, and the bottom surface temperature of the glass is $T_{w}$, which can be measured by thermocouples. Because the glass is relatively thin, it is considered that the internal temperature distribution of the glass is linear, so the above formula can be simplified as

(20) $$q=\frac{1}{2}Ac\rho h_{g}\frac{dT_{w}}{dt},$$

where *A* is taken as the unit area, the thickness of fireproof glass is 5 mm, the density is 2500 kg/m^3^, and the specific heat capacity is 0.84 kJ/(kg·K). Thus, the convective heat transfer between the fuel layer and the glass bottom can be calculated. The result is shown in the Figure 12.

In Figure 12, it can be seen that the convective heat transfer between the fuel layer and the bottom increases with the change of slope. The greater the fuel delivery, the more obvious the change of convective heat transfer with the slope. Obviously, due to the increase of slope, the spread rate and spread area of spill fire increase. It can be seen from the experimental data that when the flow is large, the bottom surface flow heat transfer energy density increases by about 20% with the increase of slope from 0° to 2°. This result shows that the heat loss by convective heat transfer between fuel layer and bottom surface has a great influence on the burning process.

#### 3.5.2. Flame Feedback Radiation Transmission

**Figure 13:**
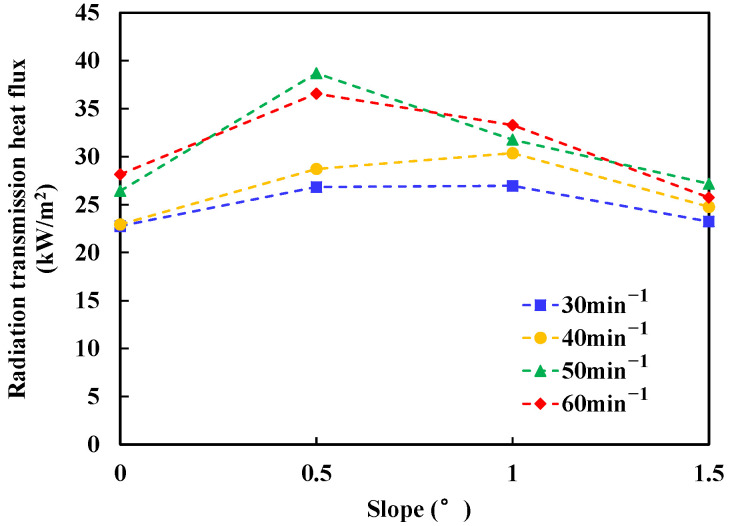
Variation of n-heptane flame feedback radiation transmission with slope.

The heat flux meter measures the radiant heat loss through the fuel layer and the glass bottom, and the measurement results are shown in Figure 13.

In Figure 13, it can be found that the transmission of the flame radiative feedback increases first and then decreases when the slope increases. It is well known that the radiative transmission is very sensitive to the liquid fuel layer [26]. According to the non-ignited experiments, the fuel layer thickness decreases obviously when the slope increases from 0° to 0.5°. As a result, the initial increase trend can be attributed to the decrease of the liquid fuel layer thickness for the large slope cases. However, when the slope continues to increase, the decreasing trend of the liquid fuel layer thickness is not obvious, as shown in Figure 4b. Meanwhile, the flame will become short and weak, which results in the lower flame radiative heat feedback and the corresponding decreasing trend for the flame radiative transmission.

#### 3.5.3. Burning Rate Calculation for Spill Fires

**Figure 14:**
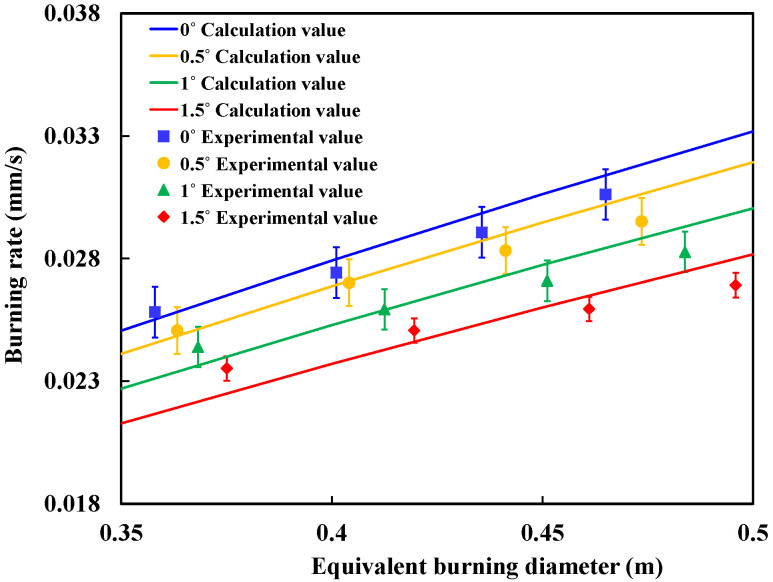
Variation of burning rate ratio of spill fire with slope.

Based on the heat transfer process, the spill fire burning rate can be calculated and the detail values are shown in Figure 14.

It can be seen that the calculation value fits the experimental results well on the flat surface (0°) and inclined surface with small slope (0.5°) in Figure 14. However, on the inclined surface with a larger slope (1° and 1.5°), the predictive values are larger than the experimental results. This is because the fuel layer thickness in non-ignited conditions is used in calculations as the fuel layer thickness in ignited conditions. However, the fuel layer thickness under ignited conditions will become smaller.

This will result in the larger calculation results. Meanwhile, the shape of the spreading surface is no longer a water droplet shape with the larger slopes. Therefore, the error caused by calculating area using Formula (4) will gradually increase with the slope.

## 4. Conclusions

In this paper, the spread and burning process of continuous spill fire leaked from point source under different slopes are studied, and the spread rate, burning rate and flame height of spill fire under different slopes are analyzed. The main conclusions are summarized:

(1) For the continuous spill fire leaked from a point source, the spread area is very sensitive to the slope. On the inclined surface, the shapes of the spread area are elliptical (under the non-ignited condition) and water droplet shaped (ignited condition). The spreading length increases obviously as the slope increases, while the spreading width decreases with the slope. Meanwhile, the total spread area will increase with the slope.
(2) The flame height decreases as the slope increases. This can be attributed to the lower burning rate, and the flame bifurcation.
(3) The steady burning rate shows a decreasing trend with the slope. The heat loss including the convection and the flame radiative transmission is the main reason behind the lower burning rate.
(4) When the slope increases, the heat convection between fuel layer and bottom increases significantly, while the feedback radiation transmission first increases then decreases.
(5) Based on the heat transfer process, a burning rate model for spill fires leaked from a point source is built and validated by the experimental data. This model can accurately predict the burning rate on different slopes.

In spill fire accidents, fuel usually spreads on a surface such as concrete rather than a glass surface. The properties of the bottom surface have a great influence on spill fires with a slope. Future studies will focus on the effect of the bottom surface for spill fires with a slope.

## Figures and Tables

**Table 1:** The relationship between peristaltic pump rotation speed and discharge rate.

Rotation Speed (rad/min)	Quality (g/s)	Volume (mL/min)
30	1.2	105.3
40	1.6	140.4
50	2.0	175.4
60	2.4	210.5

**Table 2:** Specification of the testing configurations.

Test Number	Slope (°)	Peristaltic Pump Rotation Speed (rad/min)	Test Number	Slope (°)	Peristaltic Pump Rotation Speed (rad/min)
1	0	30	11	1	50
2	0	40	12	1	60
3	0	50	13	1.5	30
4	0	60	14	1.5	40
5	0.5	30	15	1.5	50
6	0.5	40	16	1.5	60
7	0.5	50	17	2	30
8	0.5	60	18	2	40
9	1	30	19	2	50
10	1	40	20	2	60

**Table 3:** *h_m_* value under different slopes.

Slope (°)	*h_m_*—Stable Values (mm)
0	0.61
0.5	0.41
1	0.39
1.5	0.41
2	0.42

**Table 4:** Variation of burning area with slope in steady stage (m^2^).

	0°	0.5°	1°
30 rad/min	0.0897	0.1037	0.1127
40 rad/min	0.1295	0.1192	0.1558
50 rad/min	0.1458	0.1515	0.1622
60 rad/min	0.1597	0.1587	0.1646

**Table 5:** Variation of burning rate with slope in the steady stage(mm/s).

	0°	0.5°	1°
30 rad/min	0.02896	0.02507	0.02305
40 rad/min	0.02675	0.02907	0.02224
50 rad/min	0.02972	0.02859	0.02671
60 rad/min	0.03254	0.03275	0.03159

## Data Availability

Not applicable.
